# Reduced Endothelin-2 and Hypoxic Signaling Pathways in Granulosa-Lutein Cells of PCOS Women

**DOI:** 10.3390/ijms22158216

**Published:** 2021-07-30

**Authors:** Magdalena Szymanska, Ketan Shrestha, Eliezer Girsh, Avi Harlev, Iris Eisenberg, Tal Imbar, Rina Meidan

**Affiliations:** 1 Department of Animal Sciences, The Robert H. Smith Faculty of Agriculture, Food and Environment, The Hebrew University of Jerusalem, Rehovot 7610001, Israel; m.szymanska@pan.olsztyn.pl (M.S.); ketanshrestha@gmail.com (K.S.); 2 Institute of Animal Reproduction and Food Research of the Polish Academy of Sciences, Tuwima 10, 10-748 Olsztyn, Poland; 3 UK Medical Center, Department of Obstetrics and Gynecology, University of Kentucky, Lexington, KY 40536, USA; 4 Fertility and IVF Unit, Barzilai University Medical Center, Ashkelon 7830604, Israel; eliezerg@bmc.gov.il (E.G.); harlev@bgu.ac.il (A.H.); 5 Faculty of Health Sciences, Ben-Gurion University of the Negev, Beer-Sheva 8410501, Israel; 6 The Magda and Richard Hoffman Center for Human Placenta Research, Department of Obstetrics and Gynecology, Hadassah-Hebrew University Medical Center, Jerusalem 91240, Israel; IrisE@most.gov.il; 7 Hadassah Ein Kerem Medical Center, Fertility Preservation Unit, Faculty of Medicine, Hebrew University of Jerusalem, Jerusalem 9112102, Israel

**Keywords:** miR-210, *VEGFA*, *HIF-1α*, ovulation, corpus luteum

## Abstract

Granulosa-lutein cells (GLCs) from PCOS women display reduced *HIF-1α* and *EDN2* levels, suggesting their role in PCOS etiology. Here, we investigated the mechanisms involved in aberrant *EDN2* expression in PCOS, and its association with *HIF-1α*. Various *HIF-1α*-dependent factors were studied in GLCs from PCOS and compared to normally ovulating women. MicroRNA-210 (miR-210), its target genes (*SDHD* and *GPD1L*), and *HIF-1α*-responsive genes (*EDN2* and *VEGFA*) differed in GLCs from PCOS, compared with those of healthy women. Levels of miR-210—designated hypoxiamiR—and *EDN2* were reduced in the PCOS GLCs; concomitantly, *GPD1L* and *SDHD* levels were elevated. Cultured GLCs retained low *EDN2* expression and had low *HIF-1α* levels, providing evidence for a disrupted hypoxic response in the PCOS GLCs. However, *VEGFA* expression was elevated in these cells. Next, miR-210 levels were manipulated. miR-210-mimic stimulated *EDN2* twice as much as the miR-NC-transfected cells, whereas miR-210-inhibitor diminished *EDN2,* emphasizing the importance of hypoxiamiR for *EDN2* induction. Intriguingly, *VEGFA* transcripts were reduced by both miR-210-mimic and -inhibitor, demonstrating that *EDN2* and *VEGFA* are distinctly regulated. Disrupted hypoxic response in the GLCs of periovulatory follicles in PCOS women may play a role in ovulation failure, and in the reduced fertility prevalent in this syndrome.

## 1. Introduction

Polycystic ovary syndrome (PCOS) is a common reproductive and endocrine disorder affecting 5–20% of women of reproductive age worldwide [1]. It accounts for approximately 75% of the anovulatory infertility disorders [2], and is characterized by an- or oligo-ovulation; these women depend on ovulation-inducing drugs to conceive. Clinical or laboratory evidence of the appearance of hyperandrogenism and polycystic ovaries in gynecological ultrasound were described [3,4]. Current evidence suggests that granulosa cells (GCs) in the early growing follicles of PCOS women have increased proliferation [5], decreased apoptosis [6], and improper hormone production [7,8,9], which may contribute to the abnormal folliculogenesis and disordered ovulation. Previous reports have identified numerous differentially expressed genes in ovaries from women with PCOS compared with healthy controls; these genes are involved in numerous biological processes [10,11,12]. Among the differentially expressed genes was endothelin-2 (*EDN2*); we reported that *EDN2* levels were lower in the granulosa-lutein cells (GLCs) of women with PCOS [8]. This is a highly relevant finding because *EDN2*, transiently elevated around the time of ovulation [13,14], plays a crucial role in follicular rupture, ovulation, and subsequent corpus luteum (CL) formation [14,15,16]. However, the possible mechanisms underlying this aberrant *EDN2* expression in PCOS have not yet been elucidated. 

The hypoxic mediator hypoxia-inducible factor 1 alpha (*HIF-1α*) is a potent stimulus of *EDN2* transcription in the GCs of various species, including humans [14,17,18,19]. In vitro, hypoxic conditions (reduced oxygen tension, or a hypoxia-mimicking agent, such as cobalt chloride (CoCl_2_)) led to the simultaneous elevation of *HIF-1α* protein [20,21,22] and *EDN2* transcripts [14,18,22]. Vascular endothelial growth factor A (*VEGFA*) and solute carrier family 2 member 1 (*SLC2A1*) were similarly induced [22], whereas *HIF-1α* knockdown in these cells abolished their expression*,* confirming that these genes are *HIF-1α*-responsive in GCs [17,19,22]. 

MicroRNA-210 (miR-210)—designated hypoxiamiR—is another direct transcriptional target of *HIF-1α*, as reported in both non-granulosa [23,24] and granulosa cells [19]. Our previous study showed the importance of miR-210 in the *HIF-1α*-mediated upregulation of *EDN2* in human GLCs [19]. Two molecules—glycerol-3-phosphate dehydrogenase 1-like (*GPD1L*) and succinate dehydrogenase complex subunit D (*SDHD*)—were established as miR-210 targets, mediating the *HIF-1α*-stabilizing effect of this miR in GLCs [19,25,26,27]. Whether miR-210 has a distinct expression and function in the GCs of PCOS remains unknown. 

The present study was undertaken to examine hypoxic pathways in the human GLCs of PCOS. Specifically, we compared the expression of miR-210, its target genes (*GPD1L* and *SDHD*), *HIF-1α,* and *HIF-1α*-responsive genes (*EDN2*, *VEGFA,* and *SLC2A1*) in GLCs from PCOS and normally ovulating women. Additionally, to critically determine the role of miR-210, we manipulated its levels in human GLCs using miR mimics and inhibitors.

## 2. Results

### 2.1. Clinical Characteristics of PCOS and Control Patients

The demographic and clinical characteristics of PCOS and normal ovulatory women that participated in this study are presented in Table 1. The age and follicle-stimulating hormone (FSH) levels during the early follicular phase were comparable between the groups; however, the body mass index (BMI) was significantly higher in the PCOS women than in the control group made of male factor infertility (MFI) patients (27.0 ± 5.26 vs. 23 ± 5.4). The average basal luteinizing hormone (LH) levels, the LH to FSH ratio, and the serum testosterone concentrations in PCOS patients were elevated more than in the control group (7.5 ± 3.59 vs. 5.4 ± 2.77, 1.4 ± 0.87 vs. 0.9 ± 0.55, and 2.2 ± 1.18 vs. 1.5 ± 0.85, respectively).

Cycle characteristics, including estradiol (E2) levels on the day of final oocyte maturation and the number of oocytes retrieved, were similar in the two IVF groups, indicating a similarly controlled ovarian hyperstimulation protocol.

### 2.2. Altered miRs and Gene Expression Levels in Freshly Isolated GLCs from PCOS and Healthy Women

miR profiling using the NanoString analysis of freshly isolated GLCs from PCOS patients and healthy women revealed that miR-210 was among the top five miRs, clearly exhibiting significantly reduced expression in the GLCs of PCOS patients, compared with those of normally ovulating women (Table 2). Aside from miR-210, we also identified miR-4284, miR-132-3p, miR-483-3p, and miR-1973 as the most underexpressed miRs in GLCs from the PCOS group. Since we previously identified miR-210 as a regulator of *EDN2* in GLCs [19], we decided to investigate it further. MiR-210 counts were 3.2-fold lower in the GLCs from women with PCOS, compared with controls (408 vs. 1321 molecules). 

**Figure 1:**
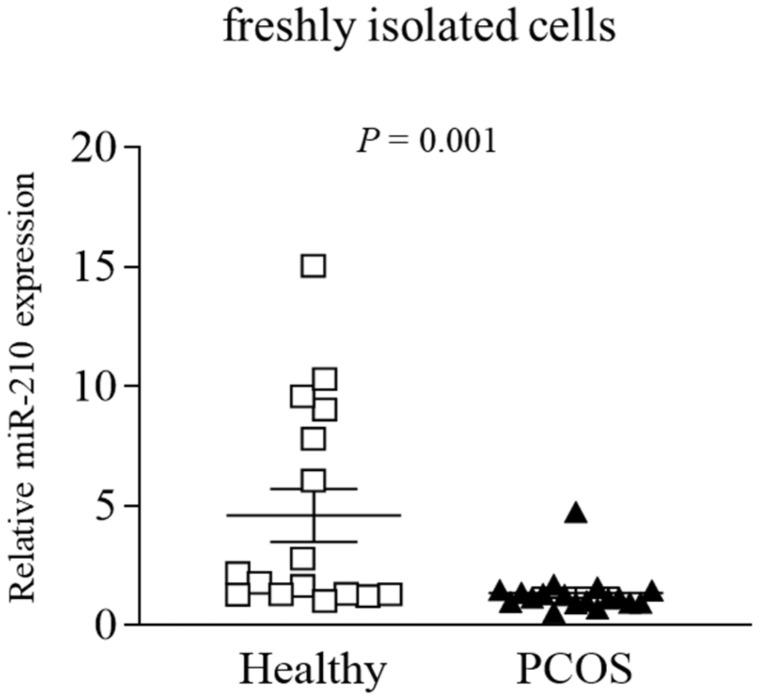
Relative expression of miR-210 in freshly isolated GLCs from PCOS and healthy normally ovulating women. miR-210 levels were measured in freshly isolated GLCs from PCOS and healthy control women by qPCR. Results are presented as a scatter plot with mean values ± SEM (n = 16 per group); squares denote values obtained in healthy controls, triangles denote values obtained in the PCOS group. The *p*-value indicates the statistically significant difference between groups.

Quantitative PCR analysis validated these findings, showing 3.4-fold lower levels of miR-210 in the GLCs from PCOS than from control women (Figure 1). 

**Figure 2:**
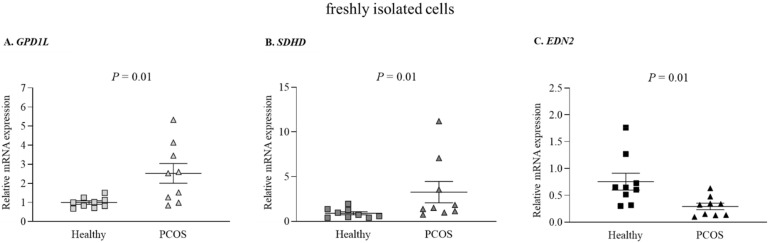
Relative expression of *GPD1L*, *SDHD*, and *EDN2* in freshly isolated GLCs from PCOS and healthy, normally ovulating women. (**A**) *GPD1L*, (**B**) *SDHD*, and (**C**) *EDN2* mRNA levels were measured in freshly isolated GLCs from PCOS and healthy control women by qPCR. Results are presented as scatter plots with mean values ± SEM (n = 9 per group); squares denote values obtained in healthy controls, triangles denote values obtained in the PCOS group. The *p*-values indicate statistically significant differences between groups.

Next, we determined the *GPD1L* and *SDHD* expression levels, confirmed to be the gene targets of miR-210 in GLCs [19]. Indeed, concomitantly with the low levels of miR-210 (Table 2, Figure 1), we found that *GPD1L* (Figure 2A) and *SDHD* (Figure 2B) transcripts were significantly elevated in the GLCs from the PCOS group, compared with a healthy control group (a 2.1- and 3.6-fold difference, respectively). Additionally, along with a lower miR-210 level, freshly isolated cells derived from PCOS women exhibited significantly reduced levels of *EDN2* (Figure 2C). 

**Figure 3:**
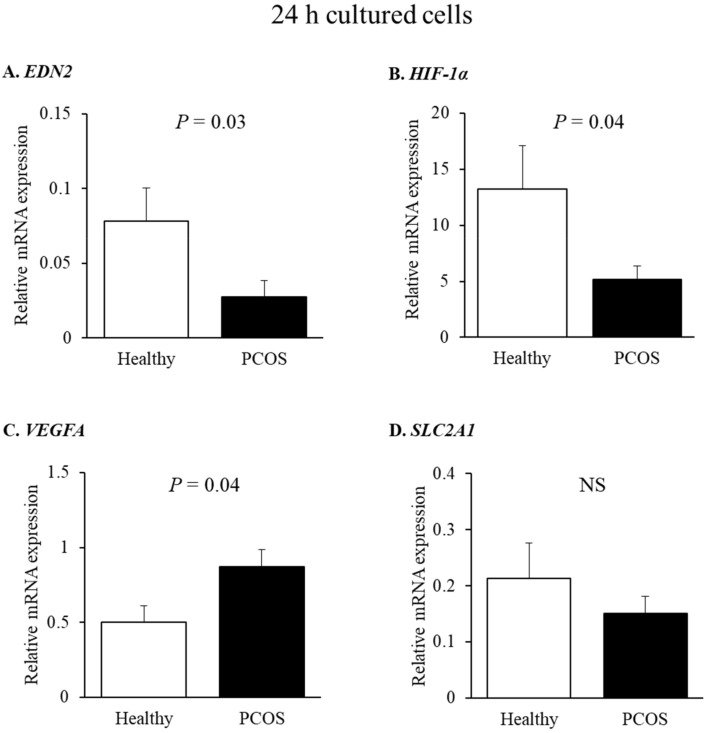
Relative expression of *HIF-1α* and selected *HIF-1α*-responsive genes in cultured GLCs derived from PCOS and healthy, normally ovulating women. GLCs derived from PCOS and healthy control women (n = 6 in both groups) were incubated with culture medium for 24 h. Then, RNA was extracted, and (**A**) *EDN2*, (**B**) *HIF-1α*, (**C**) *VEGFA*, and (**D**) *SLC2A1* levels were determined by qPCR. The results are presented as the means ± SEM. The *p*-values indicate statistically significant differences between groups. NS: non-significant.

To further substantiate these findings, we determined the expression of *HIF-1α* and several *HIF-1α*-responsive genes—*EDN2*, *VEGFA,* and *SLC2A1*—in GLCs cultured for 24 h, derived from PCOS and healthy women (Figure 3). In accordance with what was observed in the freshly isolated cells (Figure 2), the expression of *EDN2* was lower in cultured GLCs from the PCOS group (~2.6-fold less than in the GLCs of healthy controls (Figure 3A). *HIF-1α* also exhibited reduced expression in the GLCs of PCOS women (Figure 3B). Contrary to *EDN2*, *VEGFA* expression was significantly elevated in cells from PCOS women (Figure 3C). However, the expression of *SLC2A1—*another *HIF-1α*-responsive gene—remained similar in both groups (Figure 3D).

### 2.3. Effect of miR-210 on HIF-1α-Responsive Genes: EDN2 and VEGFA in Human GLCs

**Figure 4:**
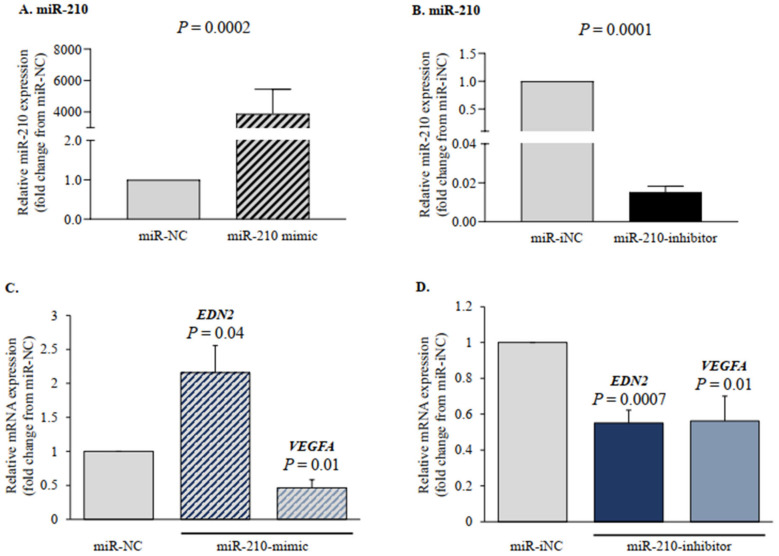
miR-210 manipulation affects the expression of *HIF-1α*-responsive genes—*EDN2* and *VEGFA*—in human GLCs. (**A**,**C**) Human GLCs were transfected with 10 nmol/L of either miR-210-mimic or miR-negative control (miR-NC). (**B**,**D**) Human GLCs were transfected with 60 nmol/L of either miR-210-inhibitor or miR-inhibitor negative control (miR-iNC). RNA was extracted at 48 h after transfection, and miR-210 (**A**,**B**), *EDN2,* and *VEGFA* (**C**,**D**) levels were determined by qPCR. The results are presented as the means ± SEM from three independent experiments. The *p*-values indicate statistically significant differences from respective NCs, designated as 1.

To better understand the role of miR-210, we manipulated its levels in human GLCs using miR-210-mimic or miR-210-inhibitor. The data presented in Figure 4A,B show that miR-210-mimic markedly elevated the miR-210 expression levels, whereas miR-210-inhibitor effectively reduced the miR-210 levels, compared with their respective negative controls. Next, we studied the role of miR-210 on *HIF-1α*-responsive genes—*EDN2* and *VEGFA*—in GLCs. The overexpression of miR-210 stimulated *EDN2* twice as much as the miR-NC-transfected cells did (Figure 4C), whereas miR-210-inhibitor had the opposite effect: it significantly diminished *EDN2* expression (Figure 4D), thus confirming that miR-210 stimulates *EDN2* in GLCs. Intriguingly, *VEGFA* transcripts were reduced by both miR-210-mimic (Figure 4C) and -inhibitor (Figure 4D).

## 3. Discussion

The present study shows that the *HIF-1α*-responsive factors in freshly isolated GLCs of PCOS patients resulted in an altered expression pattern, compared with healthy women; this suggests that a disrupted hypoxic response exists in these cells. Specifically, we found anomalous levels of miR-210, *GPD1L*, *SDHD, VEGFA,* and *EDN2* in GLCs derived from PCOS. Cultured GLCs retained the pattern of low *EDN2* expression observed in freshly isolated cells, and also exhibited low *HIF-1α* levels. In contrast to *EDN2, VEGFA* levels were higher in GLCs derived from PCOS. We found that *EDN2* expression was dependent on miR-210 and its target genes in primary cells or in miR-210-transfected cells, whereas *VEGFA* did not show such a dependency. These results suggest that reduced miR-210 expression in GLCs obtained from PCOS women upregulates *GPD1L* and *SDHD,* and destabilizes *HIF-1α*, thus reducing *EDN2*. 

One of the novel findings of this study is that the GLCs of PCOS women exhibited markedly reduced levels of miR-210, compared with cells of normally ovulating women. 

**Figure 5:**
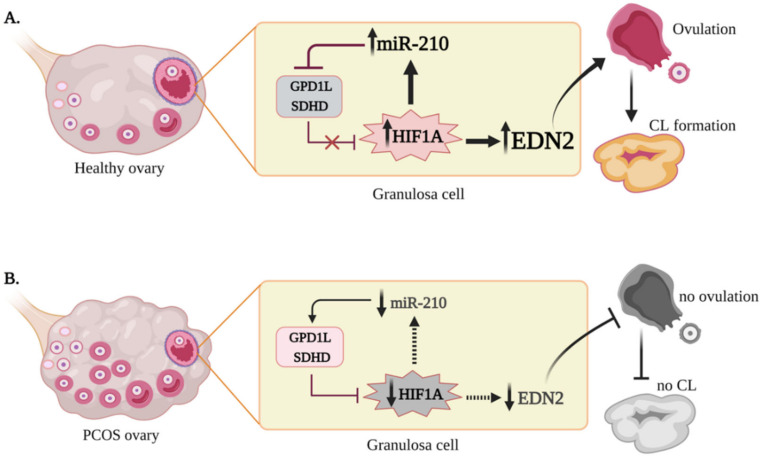
Proposed role of hypoxia-related factors in PCOS ovulatory defects, compared with normally ovulating women. (**A**) In healthy human GLCs, *HIF-1α* transcriptionally upregulates miR-210 levels which, in turn, downregulates its target genes (*GPD1L* and *SDHD*), thereby stabilizing *HIF-1α*. This feed-forward loop between miR-210, the miR-210 target genes, and *HIF-1α* enhances *EDN2* in GCs during ovulation and CL formation. (**B**) However, in the GLCs of PCOS women, the suppressed expression of *HIF-1α* leads to reduced miR-210 levels. Lower miR-210 elevates *GPD1L* and *SDHD*, which destabilize *HIF-1α* and reduce *EDN2*. Low levels of *EDN2* may cause ovulatory defects in women with PCOS. The figure was created with BioRender.com.

A positive feedback loop between miR-210, *GPD1L*, *HIF-1α*, and *EDN2* was described in our previous study [19], and this was further substantiated here (Figure 5); hypoxia-induced or overexpression of miR-210 resulted in reduced *GPD1L* and *SDHD* expression, accompanied by higher *HIF-1α* protein and *EDN2* levels in human GLCs. However, in PCOS cells, miR-210 was lower; this had functional consequences, causing reduced *EDN2* and elevated levels of *GPD1L* and *SDHD* in these cells. *GPD1L* and *SDHD* are established miR-210 targets in human GLCs [19]. Regarding the role of miR-210 in hypoxia, these two genes destabilize *HIF-1α* by acting through different mechanisms—*GPD1L* mediates the hyperhydroxylation of *HIF-1α* by increasing the activity of the prolyl hydroxylase domain (PHD) enzymes [25], whereas reduced *SDHD* increases succinate accumulation, a natural inhibitor of PHD activity [26,27]; this subsequently causes a proteosomal degradation of *HIF-1α* [28]. In fact, suppressed *GPD1L* (by either elevated miR-210 or siRNA silencing) is required for *HIF-1α* accumulation, enabling the *HIF-1α*-responsive gene *EDN2* to be stimulated [19]. However, in PCOS GLCs, this feedback loop is breached, since they express low miR-210 levels, resulting in the underexpression of *HIF-1α* and *EDN2,* compared with the cells of control women. Recently published data confirm these findings, demonstrating an abnormally lower expression of *HIF-1α* [29] and *EDN2* [8] in these cells. It is noteworthy that the positive feedback loop between miR-210, *HIF-1α*, and *EDN2* suggested here is supported by an in vivo profile of these factors: all three agents are markedly upregulated in early CL [14,15,17,30,31].

Avascular GCs survive in a hypoxic environment, and the O_2_ tension of follicular fluid negatively correlates with follicular size [32]. Such conditions support the transcription of hypoxia-dependent genes, including *EDN2*. *EDN2* has been described as a trigger of follicular rupture; it acts by constricting the smooth muscle layer around periovulatory follicles [13]. As reported for mice and rats, blocking *EDN2* action via a receptor antagonist or gene knockout inhibited follicle rupture, oocyte release, and CL formation [15,16,33]. *EDN2* may also affect follicular rupture and ovulation by other mechanisms. Since it is a vasoactive hormone, *EDN2* is likely to promote vascular changes that facilitate the ovulatory process [16]. Another HIF-regulated factor, ADAMTS-1—a matrix metalloprotease—may also participate in the remodeling of the extracellular matrix in the cumulus–oocyte complex, and in the disintegration of the follicular wall during the final stages of ovulation [34]. Owing to lagging angiogenesis, hypoxia is also prevalent during CL formation, favoring the upregulation of hypoxia-dependent genes [35]. In addition to follicular rupture, *EDN2* may facilitate early CL development by promoting angiogenesis, cell proliferation, and differentiation. For example, in cattle, *EDN2* increased GC numbers, as did PTGS2—a rate-limiting enzyme in prostaglandin synthesis, which is essential for ovulation [14]. These were also substantiated in ECE1 (EDN-converting enzyme 1) gene knockdown in bovine GCs, which resulted in reduced viable cell numbers and gene expression [14]. 

In contrast to *EDN2*, the mRNA levels of *VEGFA*—an established *HIF-1α* -dependent gene—were higher in PCOS GLCs than in healthy controls. These results are in accordance with the immunohistochemical evidence of extensive *VEGFA* staining in polycystic ovarian tissue [36,37], as well as with higher concentrations of *VEGFA* released to culture medium by the GLCs of PCOS women, compared with the non-PCOS group [38]. In addition, *VEGFA* was found to be elevated in the serum and follicular fluid of women with PCOS [29,39,40,41,42]. However, a few studies reported a reduction or no changes in the *VEGFA* levels in the GLCs of patients with PCOS [29,43]; the reason for this controversy is currently unclear. High *VEGFA* may contribute to the occurrence of PCOS by excess angiogenesis; inhibition of *VEGFA* in the rat ovaries of a PCOS model partially restored the accumulation of the small follicles observed, and reduced cyst formation, consequently improving ovulation and follicular development [44]. Both *EDN2* and *VEGFA* are dependent on *HIF-1α*; however, only the relationship between *EDN2* and *HIF-1α* is manifested in vivo around the time of ovulation [14,17,45]. *VEGFA*, which is elevated as the follicle matures, increases further during the early luteal phase, but remains elevated for the duration of the luteal phase [31,46,47]. Aside from hypoxia, there is a strong LH/hCG dependency on *VEGFA* transcription. In humans, macaques, and bovine GLCs, *VEGFA* expression [14,31,48,49,50]—as well as its secretion to the culture medium—was augmented by LH/hCG [38,41]. Of particular relevance, a strong correlation between *VEGFA* synthesis and hCG levels was observed in PCOS women, both in vivo and in vitro [38,41]. After hCG administration, PCOS women exhibited a rise in serum *VEGFA* that was not observed in the control patients [41]. Similarly, in vitro hCG treatment of GLCs derived from PCOS patients significantly increased *VEGFA* secretion; this production was greater in cells extracted from PCOS ovaries than from normal ones [38]. Because we showed an impaired hypoxic response of PCOS GLCs, we presume that an altered response to LH/hCG, rather than to hypoxia, results in increased *VEGFA,* as observed in GLCs derived from PCOS patients. However, additional research is needed in order to determine whether signals other than LH or hypoxia may be responsible for the aberrant high expression of *VEGFA* observed here in the GLCs of PCOS women.

In addition to GLCs, the follicular fluid aspirates contain non-steroidogenic cells—mainly leukocytes—to varying degrees [51,52,53]. However, although we did not remove these potentially contaminating cells from freshly isolated GLCs, we took advantage of a selective adherence of cultured cells to the surface of cell culture dishes; GLCs attach early, whereas lymphocytes—the large population of leukocytes in follicular fluid aspirates [54]—are non-adherent cells [53,55]. It should be noted that freshly isolated and cultured GLCs exhibited similar expression profiles, suggesting that the findings reported here represent the biological functions of GLCs. Nevertheless, further research using GLCs after purification steps—such as anti-CD45 magnetic immunobead, still remains to be carried out.

In summary, our study implies that the hypoxic responses of GLCs derived from women with PCOS are altered, suggesting a mechanism that may contribute to abnormal ovarian function. These findings are consistent with the notion that *HIF-1α* is a critical regulator of various ovarian processes, and that miR-210 and *EDN2* are essential *HIF-1α*-responsive factors for follicular rupture, ovulation, and CL formation. Low miR-210, causing decreased *EDN2* during the periovulatory stage, can result in the ovulation failure and reduced fertility observed in this syndrome (Figure 5). Elevated *VEGFA* in PCOS GLCs, irrespective of a lower hypoxic response, may further exacerbate the abnormal ovarian function.

## 4. Materials and Methods

### 4.1. Subjects

Forty-one women with PCOS and forty healthy, normally ovulating women were enrolled in this study. PCOS was diagnosed according to the Rotterdam revised criteria [3]. The control patients entered the IVF program with non-ovarian indications, which were limited to male factor infertility (MFI). For both the PCOS and control groups, women were subjected to the GnRH antagonist protocol [56]. In brief, controlled ovarian stimulation was initiated on day 2 or 3 of a spontaneous cycle. An initial dose of 150–225 IU recombinant FSH (rFSH; Gonal-F (Merck Serono, Darmstadt, Germany)), Puregon (MSD, Hertfordshire, UK), or highly purified hMG (Menopur (Ferring Pharmaceuticals, Saint-Prex, Switzerland)) was administered. From day 6 onward, the gonadotropin dose was estimated according to the serum E2 levels and a transvaginal ultrasound scan. When a leading follicle reached 13–14 mm, a GnRH antagonist (Cetrotide (Merck Serono) or Orgalutran (MSD)) was administered at 0.25 mg/d. Final oocyte maturation was triggered using 250 µg recombinant hCG (rhCG; Ovitrelle (Merck Serono)) as soon as the mean diameters of two follicles were ≥18 mm. Oocyte retrieval was scheduled 36 h after hCG injection. All accessible follicles were harvested, and oocytes were collected from follicular aspirates and subjected to fertilization. The residual follicular fluid aspirates containing GLCs were collected for further investigation. 

### 4.2. Experiment Protocol

GLCs were obtained from follicular aspirates as previously described [57]. Briefly, the aspirates were centrifuged (3 min at 3000× *g*) and erythrocytes were removed using an ammonium-chloride–potassium buffer (0.15 mol/L NH_4_Cl, 1.0 mmol/L KHCO_3_, and 0.1 nmol/L EDTA). After having been washed with phosphate-buffered saline (PBS), cells were counted in a hemocytometer. A fraction of the freshly isolated cells (n = 16 from each group of patients) was taken and stored at −80 °C for further RNA extraction. 

For in vitro experiments, cells were placed in 12- or 6-well plates (1.5 × 10^5^ cells/well) and cultured in a DMEM/F12 1:1 (*v*/*v*) nutrient mixture containing 10% fetal calf serum (FCS), 2 mM L-glutamine, and 100 mg/mL penicillin/ streptomycin (Biological Industries, Kibbutz Beit HaEmek, Israel). Cultures were maintained in a humidified 95% air 5% CO2 mixture at 37 °C. Following 24 h of incubation, cells were washed with PBS and collected for subsequent RNA isolation, or they were subjected to transfection as detailed below.

### 4.3. Cell Transfection

miR-210-mimic, miR-210-inhibitor, and the corresponding negative controls (miR-NC or miR-iNC, respectively) were purchased from Ambion (Thermo Fisher Scientific, Waltham, MA, USA) and Bioneer (Daejeo, Korea). Cells were transfected with miR-210-mimic (10 nmol/L), miR-210-inhibitor (60 nmol/L), or their respective NCs in 1% FCS for 24 h using Lipofectamine RNAiMAX reagent (Thermo Fisher Scientific) according to the manufacturer’s protocol. Forty-eight hours after transfection, the cells were harvested and total RNA was extracted.

### 4.4. Quantitative PCR Analysis of mRNA and miRNA

Total RNA was extracted using the TRI Reagent (Molecular Research Center, Cincinnati, OH, USA) in accordance with the manufacturer’s instructions. cDNA was synthesized from the total RNA (1000 ng) by using the qScript cDNA synthesis kit (Quantabio, Beverly, MA, USA). miRNA cDNA was obtained from the purified total RNA (700 ng) using the qScript microRNA Synthesis Kit (Quantabio). Quantitative polymerase chain reaction (qPCR) for mRNA expression was performed with LightCycler 480 SYBR Green I Master (Roche Diagnostics, Indianapolis, IN, USA) and for miR-210 with PerfeCTa SYBR Green SuperMix, Low ROX (Quantabio), as previously described [19,57]. Each PCR reaction was performed in duplicate. The PerfeCTa microRNA assay included Universal Primer, miR-210 Primer, and SNORD44 as positive control primers (Quantabio). The expression levels were normalized to beta-actin (*ACTB;* for mRNA) and ribosomal protein S18 (*RPS18;* for miRNA). The sequences of primers used for qPCR are listed in Table 3. The threshold cycle (Ct) values of each sample were generated, and the relative expression was calculated as 2^-ΔCt^=2^-(Ct target gene − Ct housekeeping gene)^ [58].

### 4.5. MiRNA Expression Profiling

A global analysis approach was employed to reveal the miRNA expression profile using the nCounter miRNA expression assay at NanoString Technologies. About 800 human miRNAs were simultaneously assayed (miRBase v.18). A total of 100 ng of total GLC RNA pooled either from a control group (n = 8) or from patients with PCOS (n = 8) was assayed separately in technical duplicates using the Human nCounter miRNA Assay 2.0 Kit (NanoString Technologies, Seattle, WA, USA), following the manufacturer’s instructions. Briefly, mature miRNAs were ligated to a species-specific tag sequence (miRtag) via a thermally controlled splinted ligation. After enzymatic purification of unligated miRtags, prepared samples were hybridized with an nCounter Human miRNA Expression Assay CodeSet overnight at 65 °C. Unhybridized CodeSet was removed via automated purification performed on an nCounter Prep Station, and the resulting target–probe complexes were deposited and bound to an imaging surface as previously described [59]. Reporter counts were tabulated for each sample using the nCounter Digital Analyzer, and output as raw data was subsequently imported. 

### 4.6. Statistical Analyses

Statistical analyses were performed using GraphPad PRISM v. 6.0 (GraphPad Software, Inc., San Diego, CA, USA). Student’s *t*-test with a two-tailed distribution, with two samples equaling variance was conducted. When appropriate, a non-parametric analysis was conducted using the Mann–Whitney U test. Numerical data are reported as the mean ± standard deviation (Table 1) or the mean ± standard error of the mean (SEM). A *p*-value < 0.05 was considered statistically significant. 

## 5. Conclusions

Altered hypoxic responses and angiogenic potential in GLCs affect follicle development and maturation in human ovaries. Dysregulated hypoxic responses can lead to abnormal ovulation and, eventually, infertility, as commonly observed in PCOS patients. This study, together with our previous report [19], identified the molecular makeup of the hypoxic pathway in GLCs, highlighting the role of miR-210. miRs can be detected in bodily fluids such as blood plasma, saliva, urine, and follicular fluids, and may thus serve as biomarkers for cellular activity [60,61,62,63]. It is therefore anticipated that miR-210 levels could serve as a novel diagnostic tool, and also as a new therapeutic target to treat anovulation in PCOS patients.

## Figures and Tables

**Table 1:** Demographic and clinical characteristics of the study participants.

	PCOS (n = 41)	Control (MFI; n = 40)	*p*-Value
Age (years)	27.8 ± 4.1	27.6 ± 4.8	NS
BMI (kg/m^2^)	27.0 ± 5.26	23 ± 5.4	0.001
Serum early follicular FSH (IU/L)	5.7 ± 1.86	6.2 ± 2.26	NS
Serum early follicular LH (IU/L)	7.5 ± 3.59	5.4 ± 2.77	0.009
LH:FSH ratio	1.4 ± 0.87	0.9 ± 0.55	0.007
Serum total testosterone (nmol/L)	2.2 ± 1.18	1.5 ± 0.85	0.006
Peak E2 (pmol/L)	7124.0 ± 3511	6965 ± 3663	NS
Oocytes retrieved	12.9 ± 8.0	14.3 ± 5.6	NS

PCOS: polycystic ovary syndrome; MFI: male factor infertility; BMI: body mass index; FSH: follicle-stimulating hormone; LH: luteinizing hormone; E2: estradiol; NS: non-significant; values are reported as the mean ± standard deviation.

**Table 2:** The top five miRNAs with decreased expression in granulosa-lutein cells from patients with PCOS, compared to control/normally ovulating women. The miRNA expression profile was carried out using the nCounter miRNA expression assay from NanoString Technologies (see Section 4).

miRNA	Molecule Count		Fold Change	*p*-Value
	PCOS	Control		
hsa-miR-4284	62	262	–4.2	0.009
hsa-miR-132-3p	2193	7273	–3.3	0.018
hsa-miR-210	408	1321	–3.2	0.025
hsa-miR-483-3p	233	1022	–3.1	0.032
hsa-miR-1973	63	185	–2.9	0.018

**Table 3:** Sequences of primers used for qPCR.

Gene Name	Sequence (5’-3’)	Accession No.
*GPD1L*	F: GATGCAGACACTGTTGAACTC R: AGGTGGCTGTAGACACTTGG	NM_015141 [19]
*SDHD*	F: TCCTTGCTCTGCGATGGAC R: GCTTTGCAGATGCCCACAT	NM_003002 [19]
*EDN2*	F: GCCAGCGTCCTCATCTAT R: GCCGTAAGGAGCTGTCTGTTC	NM_001956 [19]
*VEGFA*	F: ATCGAGACCCTGGTGGACA R: CCTCGGCTTGTCACATCTGC	NM_001025366 [22]
*HIF-1α*	F: ACTCATCCATGTGACCACG R: TAGTTCTCCCCCGGCTAG	NM_001530 [19]
*SLC2A1*	F: CGCTTCCTGCTCATTAACCG R: CCTTCTTCTCCCGCATCAT	NM_006516 [22]
*ACTB*	F: CGGGACCTGACGGACTACCTC R: GCCATCTCCTGCTCGAAGTCC	NM_001100 [19]
*RPS18*	F: CACCAAGAGGGCGGGAGA R: CTTCTTCAGTCGCTCCAGG	NM_022551 [19]

Abbreviations—F: forward; R: reverse.

## Data Availability

The data presented in this study are available on request from the corresponding author. The data are not publicly available due to their containing information that could compromise the privacy of research participants.
